# The Workmen's Compensation Act

**Published:** 1899-03-11

**Authors:** 


### THE WORKMEN'S COMPENSATION ACT.

In discussing the fees which ought to be paid to house surgeons under the Workmen's Compensation Act, the *Lancet* points out that "if a house surgeon is asked for an opinion on a case he is entitled to a fee. . . . He should not make it an exorbitant fee, but one as commensurate as he can make it with the particular demand on his time and the amount of compensation at stake," and goes on to say that the house surgeon should give what help he can to the practitioner who comes to examine the case, and should always be paid a fee, and that this fee should range from 10s. 6d., according to the demands made upon his time. A scale, it is said, has been adopted at Guy's Hospital according to which 10s. 6d. is asked for a short consultation, £1 1s. for a longer one, and £2 2s. for a consultation and written report. This seems fair and proper, and we think that where a surgeon, appointed in accordance with the Act, requires a consultation with a house surgeon, some such scale as the above should be adhered to.

